# Uptake of iron from ferrous fumarate can be mediated by clathrin-dependent endocytosis in Hutu-80 cells

**DOI:** 10.3389/fmolb.2025.1460565

**Published:** 2025-01-27

**Authors:** Agata Tarczykowska, Per Malmberg, Nathalie Scheers

**Affiliations:** ^1^ Division of Food and Nutrition Science, Department of Life Sciences, Chalmers University of Technology, Goteborg, Sweden; ^2^ Division of Chemistry and Biochemistry, Department of Chemistry and Chemical Engineering, Chalmers University of Technology, Goteborg, Sweden

**Keywords:** DMT1, endocytosis, iron, Hutu-80, Caco-2, ferrous fumarate, uptake

## Abstract

Iron uptake in the intestinal epithelium is associated with transport of ferrous iron via the DMT1 transporter (SLC11a2; NRAMP2). In later years, uptake of iron from complex sources, such as nanoparticles, has been found to be mediated through endocytosis. Here we propose that iron from the simple salt ferrous fumarate, a common iron supplement, can be absorbed by clathrin-mediated endocytosis. We used siRNA to silence DMT1 transporter expression, pharmacological inhibition of endocytosis, and Time-of-Flight Secondary Ion Mass Spectrometry (ToF-SIMS) to show that iron uptake from ferrous fumarate can be mediated by both transport via DMT1 and by clathrin-dependent endocytosis in Hutu-80 cells. Iron uptake (ferritin L) from ferrous fumarate (0.5 mM, 24 h) in DMT1 silenced cells was significantly decreased (60% ± 11%) in comparison to iron controls while a 1-h dose of ferrous fumarate (0.5 mM) significantly decreased ferritin L formation in the presence of the clathrin inhibitor chlorpromazine (61% ± 10%, in post-confluent cells and 37% ± 9% in non-confluent cells). A pilot showed a similar trend for Ferritin (H) levels (confluent cells) and for total cellular iron load (non-confluent cells). ToF-SIMS analysis revealed diminished membrane-associated iron load in endocytosis-inhibited ferrous fumarate treated cells. The reported results support a clathrin-mediated endocytosis mechanism for uptake of iron from ferrous fumarate in addition to iron uptake by DMT1. More studies are needed to understand what determines which uptake mechanism are employed and to which extent.

## 1 Introduction

### 1.1 Luminal uptake and basal export of iron in human intestinal cells

Iron transport across the intestinal border into the portal blood stream is mediated and regulated by different mechanisms. Iron import at the luminal side (apical surfaces of the columnar cells) is majorly depending on the iron form presented to the absorptive epithelial cells. An iron transporter for ferrous iron (Fe^2+^), the divalent metal transporter 1 (DMT1; NRAMP2; SLC11a2) (Canonne-Hergaux et al., 1999; Gunshin et al., 1997) has been considered to be the key mediator in iron uptake from non-heme iron-containing foods and supplements. A membrane-integrated reductase, Dcytb, may aid in the reduction of ferric iron to ferrous iron (McKie et al., 2001) preceding DMT1 transport. In humans, DMT1 exists in four different isoforms, varying at the C terminus (IRE+ and IRE−), or at the transcription site of the N terminus. The isoforms that contain IRE (iron responsive elements) are regulated by iron regulatory proteins (IRPs) and are thought to be of greatest importance for Fe^2+^ uptake (Laura, 2016). DMT1 also transports other divalent metal ions across the luminal plasma membrane but has shown high affinity for Fe^2+^ in human cells (Wolff et al., 2018). In addition to direct iron transport by DMT1, other luminal routes have been indicated to play roles for human intestinal uptake of iron, e.g., another metal transporter, the transmembrane protein Zrt- and Irt-like protein 14 (ZIP14), belonging to the zinc transporter family (Scheiber et al., 2019). Furthermore, it has been observed that larger molecules containing iron, such as ferritin or ferritin-mimicking molecules can be absorbed by endocytic routes (Perfecto et al., 2018; Antileo et al., 2013; Kalgaonkar and Lonnerdal, 2009). Pereira and colleagues investigated the uptake route of ferrihydrite-like nanoparticles, which were observed to be internalized into Caco-2 cells and the internalization was inhibited by Chlorpromazine suggesting they were transported via clathrin-mediated endocytosis (Pereira et al., 2013). They have also described later involvement of DMT1 in the acquisition of iron from the endosomes/lysosomes, suggesting that these two pathways may be interdependent. On a similar note, Perfecto and colleagues showed that transport of ferric phosphate nanoparticles into enterocytes is dependent on clathrin-mediated endocytosis as well as micropinocytosis (Perfecto et al., 2017). Once inside the cell, iron is stored in ferritin, an intracellular iron-storage protein composed of 24 subunits, consisting of both Light (L) and Heavy (H) chains. The L and H subunits have different roles, the H chains contain ferroxidase activity sites, while the L chains provide the electrons for the oxidation of ferrous iron into ferric iron, which is the stable storage form. Both subunits independently regulate iron transport (Sammarco et al., 2008). Ferritin (L and H) mRNA are IRE+ and is regulated by IRP:s. Additionally, ferritin H chains are regulated by hepcidin, an extracellular hormone that controls iron export and systemic iron levels, by binding to the primary iron exporter, ferroportin (Nemeth and Ganz, 2021). In summary, the present landscape implies that soluble iron is absorbed in its ferrous or possibly ferric form and that iron as constituent of proteins or particles can be absorbed by cellular endocytosis of varying types.

### 1.2 Caco-2 and Hutu-80 cells as models in iron uptake and bioavailability studies

In nutritional and pharmacological research, human intestinal cell models are used to study, e.g., drug or food uptake, transport and signaling. E.g., the combined simulated digestion/Caco-2 cell model, developed by Glahn et al. (1996) for iron uptake studies has become a popular method for iron absorption studies in which post-confluent Caco-2 cells (14 days in culture) have been validated to predict trends in iron bioavailability to humans by means of measuring ferritin production (Yun et al., 2004). It has been established that ferritin production predicts iron availability in the Caco-2 cells (Glahn et al., 1998) and as such ferritin is a common marker for iron uptake (Pereira et al., 2013; Perfecto et al., 2017; Viadel et al., 2007; Gupta et al., 2020). The standard is to use commercial serum-ferritin ELISA kits, which measure the ferritin L subunit. Caco-2 cells are also widely used for mechanistic studies of iron uptake in both nonconfluent (undifferentiated, colonic characteristics) and post-confluent cells (duodenal characteristics) without a digestion step (Scheiber et al., 2019; Pereira et al., 2013; Perfecto et al., 2017; Alvarez-Hernandez et al., 1998). Another human intestinal cell line, Hutu-80 (duodenal origin), not as widely used or validated for bioavailability studies as Caco-2 cells but is also used in mechanistic investigations of iron transport (Latunde-Dada et al., 2014; Zhang et al., 2003). The advantage of using the Hutu-80 cell line has sometimes been described to be due to a higher expression of the reductase DcytB (aiding in iron uptake by the reduction of ferric iron (Fe^3+^) to ferrous iron (Fe^2+^)) in comparison to Caco-2 cells, in which at least one study showed that Dcytb expression in Caco-2 cells was below the detection threshold (Solomou Solomis et al., 2013). Although Scheers and colleagues have observed detectable levels of Dcytb in Caco-2 cells (Scheers and Sandberg, 2008).

### 1.3 The present study

In the present study, we used siRNA (*Silencer®* Select; S9708 and S9709; Invitrogen) to silence DMT1 expression in Hutu-80 cells to establish if iron from ferrous fumarate can be absorbed in the absence of the transporter and incorporated into ferritin once inside the cells. The method validation was done on the protein level, translation efficiency, instead of transfection efficiency (as done by the manufacturer), since we were interested in the level of transporters and not their precedents. The correlation between DMT1 mRNA and protein levels can be influenced by several factors (Wang et al., 2021; Okazaki et al., 2012; Foot et al., 2008). We undertook the experiments in both non-confluent cells (48 h in culture) or in post-confluent cells (14 days in culture) since both setups are commonly used. To establish if iron was absorbed without the possibility of clathrin-mediated endocytosis, we used an inhibitor, Chlorpromazine (Merck, Darmstadt, Germany) that exerts its action by anchoring the clathrin and adaptor protein 2 (APO2) complex, preventing the assembly and disassembly of clathrin lattices in the endosomal invaginations and thereby inhibiting endocytosis (Wang et al., 1993). We did not focus on macropinocytosis or caveolae-mediated endocytosis in the present study since we have previously observed that the inhibitors Dimethyl amiloride and Filipin had no effect on ferritin levels in ferrous fumarate-treated Hutu-80 cells. In addition to estimating iron uptake via cellular ferritin (L) production, we estimated ferritin H levels by Western blot in post-confluent Hutu-80 cells. For non-confluent cells, we also measured total cellular iron load by Graphite furnace atomic absorption spectroscopy (GFAAS). Furthermore, we used ToF SIMS analysis to measure the membrane-associated iron load and cellular localisation to complement the ferritin data. The main aim was to investigate if iron from ferrous fumarate, speculatively in the form of a precipitate, and thus unavailable for DMT1, could be absorbed by other means.

## 2 Methods

### 2.1 Cell line

The duodenal adenocarcinoma cell line Hutu-80 (ATCC® HTB40) was used in the present study. For continuous culture, the medium was Minimum Essential Medium (MEM; Gibco, Thermo Fisher Scientific, Rockford, IL, United States) supplemented with heat inactivated fetal bovine serum (FBS; 10%; Gibco), L-glutamine (2 mM; Gibco), and Normocin (0.1 mg/mL; Invivogen, Toulouse, France). The cells were incubated at 37°C/CO_2_ (5%) and the medium was replaced with fresh media every two to 3 days. The cells were passaged when the confluency was 70%–80% (trypsin-EDTA 0.05%; Gibco).

### 2.2 DMT1 silencing experiments

The cells were seeded in 24-well plates (Corning Life Sciences, Lowell, MA, United States) at 50,000 cells/well. The medium used for the silencing experiments was either Opti-MEM™ or MEM-FBS (5%), Normocin (0.2%). The cells were grown for 24 h (or 96 h for confluent cells), before the siRNA mixes were added. In the very first experiments, a positive control (GAPDH silencer-extensively validated in many cell lines) and negative control (siRNA not targeting any gene product) were used to verify that the method was working. Silencer® Select siRNA (9708 or 9709; 5–750 pmoles) was prepared by mixing it with medium (total 25 μL) and incubated for 10 min. Transfection reagent, Lipofectamine™ 3000 (Invitrogen™; 0.75 μL–3 μL) was mixed with medium and incubated for 10 min as well. After 10 min both mixtures were mixed in one Eppendorf tube and incubated together for 15 min. Cellular protein levels were used to estimate how much siRNA should be used. In the next step, the cells were incubated with the prepared siRNA (50 μL + 450 μL medium) for 24 h and after that time the iron compounds (at [Fe] = 500 µM) were added for 24 h more. When the incubation time was over, the cells were washed with DPBS (Dulbecco’s Phosphate Buffered Saline without calcium and magnesium; GE Healthcare Life Sciences, Logan, UT, United States) and lysed with cold RIPA buffer (100 μL, Sigma-Aldrich, St. Louis, MO, United States) supplemented with protease inhibitors (Thermo Fisher Scientific, Rockford, IL, United States). The lysates were collected and frozen in −80°C until analysis.

### 2.3 SDS-PAGE and Western blot

Cell lysates, (7.5 μg of total protein for detection of DMT1, and 20 μg for ferritin-H), were diluted in 4 × Laemmli sample buffer with 2-mercaptoethanol and loaded on TGX-gels (Bio-rad). For samples detecting DMT1, boiling prior to loading was omitted; for ferritin H detection, boiling was conducted for 5 min at 95°C [according to (Tsuji, 2020)]. The gels were run in Tris/glycine/SDS buffer at 200 V. After electrophoresis, the separated proteins were blotted to PVDF membranes using the Trans-Blot Turbo system and the 3-min protocol (Bio-rad). After the proteins were transferred, the blots were incubated in blocking buffer (Biorad, EveryBlot Blocking Buffer) at room temperature for 1 h. The primary antibodies, Rabbit-anti-human-NRAMP2 (Alpha Diagnostics, San Antonio, TX, United States) was diluted in blocking buffer (0.2 μg/mL; C9483, Sigma-Aldrich), and Rabbit-anti-human ferritin heavy chain (Abcam, Cambridge, United Kingdom) was diluted in blocking buffer (0.5 μg/mL; #12010020, Bio-rad). The membranes were incubated for 2 h at room temperature. After washing, the blots were incubated with secondary antibody (Goat-anti-rabbbit-HRP; Bio-rad) at 0.5 μg/mL) and StrepTactin-HRP conjugate (1 μL/10 mL) for 1 h. After washing in PBS tween, a solution of luminol and peroxide buffer (Bio-rad) was added to the blots and the bands were detected by the ChemiDoc™ XRS + system (Bio-rad) and analyzed with the software Image Lab™ 3.0.1 (Bio-rad).

### 2.4 Inhibition of endocytosis

Hutu-80 cells were seeded in 12-well plates (Corning Life Sciences, Lowell, MA, United States), San Francisco, CA, United States) at 200,000 cells/well for 48 h or 14 days. On the day of the experiment, the medium was changed from MEM-FBS (10%), Normocin (0.2%) to MEM-FBS (5%), Normocin (0.2%) and the cells were supplemented with chlorpromazine (10 or 100 μM) for 30 min. After 30 min the medium was supplemented with iron solutions, except for controls, at [Fe] = 500 µM and further incubated for 60 min. The medium was replaced with MEM-FBS (5%), and the plates were returned to the incubator for further 22.5 h. The medium was aspirated, and the cells were washed with PBS (Dulbecco’s Phosphate Buffered Saline without calcium and magnesium; Cytiva HyClone, Emeryville, CA, United States) and lysed in cold RIPA buffer (200 µL for 12-well plates; Sigma-Aldrich, St. Louis, MO, United States) with added protease inhibitors (Thermo Fisher Scientific, Rockford, IL, US). The lysates were collected and frozen in −80°C until analysis.

### 2.5 Iron compounds

Ferric sodium EDTA hydrate, Ferrous fumarate and ferrous sulfate were purchased from Sigma-Aldrich (St. Louis, MO, United States). Iron stock solutions ([Fe] = 5 mM) were prepared fresh before each experiment and the iron content was verified by AAS.

### 2.6 Microwave digestion and atomic absorption spectroscopy (AAS)

Iron stock solutions were digested by microwave digestion. Each stock solution (1 mL) was mixed with ultrapure water (7 mL) and concentrated nitric acid (1.5 mL; trace metal grade; Fisher Scientific, Loughborough, UK) and concentrated hydrochloric acid (0.35 mL; trace metal grade; Fisher Scientific, Loughborough, UK) in Teflon microwave digestion vessels. The microwave digestion was conducted in a microwave lab station (Ethos Plus, Milestone; Sorisole, Italy) at 180°C for 15 min. The total Fe content was measured by atomic absorption spectroscopy (AAS; Agilent 240/280 Series AA spectrometer, Santa Clara, CA, United States).

### 2.7 Graphite furnace atomic absorption spectroscopy (GFAAS)

Standard solutions were prepared by diluting the purchased standard (certified 1000 ± 4 mg/L of iron in nitric acid (Supelco, 16596-250 mL) in milli-Q water. All measurements were carried out on cell lysates in RIPA buffer (Sigma-Aldrich, St. Louis, MO, United States) supplemented with protease inhibitors (Thermo Fisher Scientific, Rockford, IL, US) using graphite furnace atomic absorption spectroscopy (GF-AAS, Agilent 200 series AA with GTA 120, United States, Santa Clara) using palladium as chemical modifier of the furnace. Measurements were carried out at 248.3 nm, using an iron-hollow cathode lamp (current 5 mA); the slit width was 0.2 nm. The sample volume used was 15 μL. All measurements were performed using the optimized temperature program listed in Supplementary Table S1.

### 2.8 Ferritin (L) production as estimate of iron load

Aliquots of cell lysates were analysed for ferritin (# EIA 1872, DRG International Inc., CA, US) according to the manufacturer’s protocol and normalised to total protein content of each well (BCA assay, Pierce, Chicago, IL, US).

### 2.9 Cellular iron content analysis by ToF-SIMS

Cells were cultured in 12-well plates on round 1.2 mm cover glasses, 50,000 Hutu-80 cells were seeded in each well and incubated with ferrous fumarate (60 min) after 48 h in culture and pretreated with either siRNA or chlorpromazine. Before analysis, the cells were washed in ammonium formeate to remove excess metal-containing medium and then freeze-dried to conserve the cellular structure. ToF-SIMS analysis was performed using a TOF.SIMS 5 instrument (ION-TOF GmbH, Münster, Germany). The instrument was equipped with a 30 keV Bi_3_^+^ cluster ion gun as the primary ion source and a 20 keV Argon gas cluster ion source (GCIB) for sputtering (Bastos et al., 2020). Depth profiling was performed using the Ar source at 10 keV using Ar_1500_^+^ ions at 5 nA. The samples were analyzed with the primary ion beam in the delayed extraction mode, using a pulsed primary ion beam (Bi_3_^+^, 0.1 pA) with a focus of approximately 100 nm and with a mass resolution of at least M/ΔM = 4000 fwhm at *m/z* 500. Depth profiling was performed in the interlaced mode (1 shots per pixel, 1 frame per sputter cycle, 256 × 256 pixel) using a crater area of 300 × 300 μm^2^ whilst analyzing an area of 60 × 60–100 × 100 μm^2^ using the primary ions. The ratio between the analyzed area and the sputtered area was >2. All spectra, images and depth profiles were acquired and processed with the Surface Lab software (version 7.3, ION-TOF GmbH, Münster, Germany). Low energy electrons were used for charge compensation during analysis.

### 2.10 Statistics

Microsoft® Excel (Redmond, WA, United States) was used for plotting the data in bar graphs. Sample Standard Deviation and Independent Samples *t*-test for two different groups were calculated using the SPSS software (IBM SPSS Statistics version 29.0.1.1 (244). Data are presented as means of 2-4 independent cell experiments conducted at separate occasions ± Sdev. Differences were considered significant at p < 0.05.

## 3 Results

### 3.1 DMT1 silencing reduced ferritin production in ferrous fumarate-supplemented Hutu-80 cells

**FIGURE 1:**
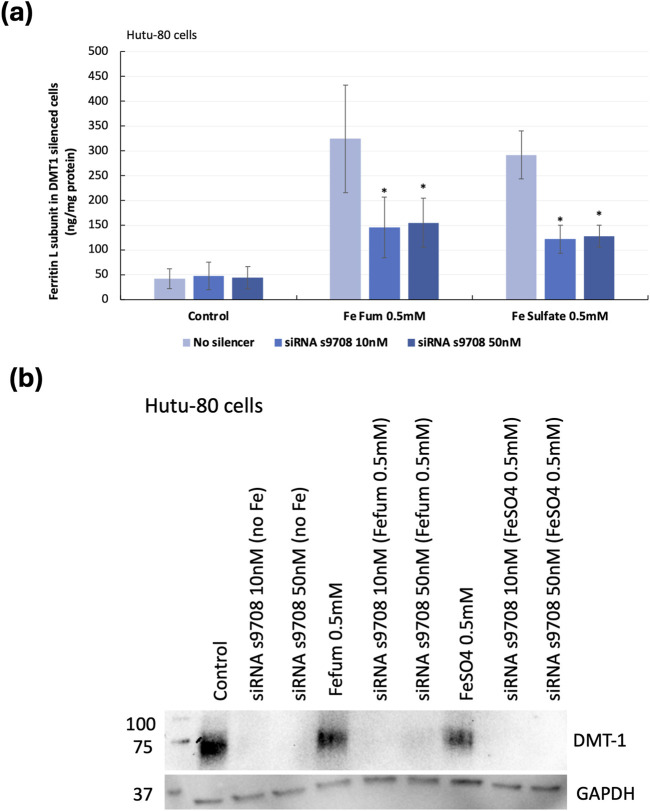
**(A)** Indirect iron load (ferritin L) in DMT1-silenced Hutu-80 cells incubated with iron for 24 h, presented as ng/mg total cell protein. Data are means ± Sdev, n = 3 separate experiments. An asterisk indicates a significant difference from the iron control (p < 0.05). **(B)** Western blot of DMT1 protein expression in non-confluent Hutu-80 cells, treated with Silencer® Select siRNA (s9708) and iron salts. 7.5 μg of total protein was loaded to each well. DMT1 exposure time: 60 s and GAPDH exposure time was 10 s.

Non-confluent DMT1-silenced Hutu-80 cells (24 h post-transfection) were incubated with iron solutions (ferrous fumarate 0.5 mM and ferrous sulfate 0.5 mM) for 24 h. To evaluate if the reduction in iron transporters decreased iron uptake, ferritin (L) levels were measured, Figure 1A. Silencing DMT1 (s9708; 10 nM) caused significant decreases in ferritin production (60% ± 11%, p = 0.00009, with ferrous fumarate 0.5 mM, 24 h and 65% ± 12%, p = 0.0002 with ferrous sulfate, 0.5 mM, 24 h). Western blot analyses, Figure 1B (representative blot), showed that DMT1 silencing efficiency (protein level) in Hutu-80 cells, supplemented with 0.5 mM Fe fumarate and treated with 10 nM S9708; MEM-FBS (5%) was: 94% ± 9%, p = 0.004. Hutu-80 cells supplemented with 0.5 mM ferrous sulfate and silenced with 10 nM siRNA s9708, the silencing efficiency reached: 98% ± 1%, p = 0.00009. There was no significant difference in silencing efficiency between the s9708 control and/or the iron treatments; on average the silencing efficiency across all iron-treated cells was 95.3% ± 3.86% (n = 8 experiments) in Hutu-80 cells.

### 3.2 Pharmacological inhibition of clathrin-mediated endocytosis in Hutu-80 cells

**FIGURE 2:**
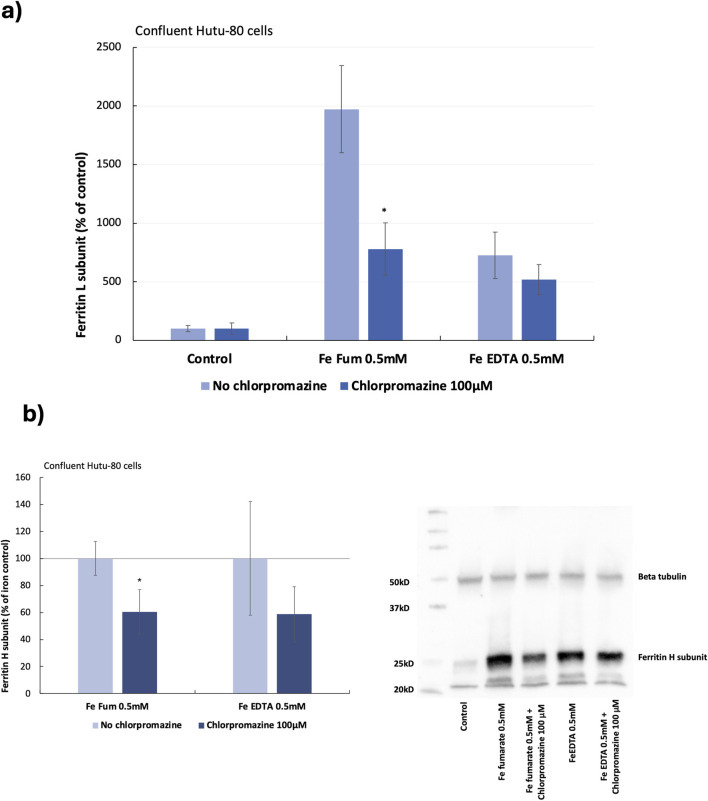
**(A)** Ferritin (L) production in post-confluent Hutu-80 cells (14 days in culture) pre-treated with the clathrin inhibitor Chlorpromazine (100 μM; 30 min) followed by a 60-min incubation of iron compounds. Data were normalized to total protein in each well and are presented as percentage of untreated control (No Fe) ± Sdev, 6 cell replicates. An asterisk indicates a significant difference from the iron control, p < 0.5. **(B)** Western blot data of ferritin H levels in post-confluent cells, data are percentage of the iron control ± Sdev, n = 4. The signal was captured by The ChemiDoc XRS+ and intensities were analyzed with the Image Lab software (BioRad).

**FIGURE 3:**
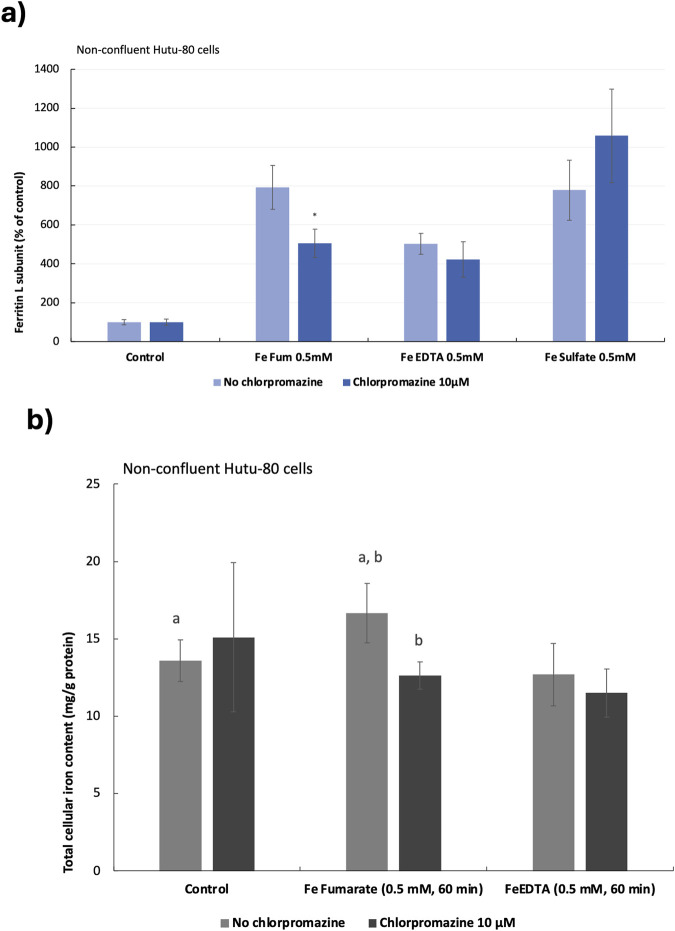
**(A)**, Ferritin production in non-confluent Hutu-80 cells pre-treated with the clathrin inhibitor Chlorpromazine (10 μM; 30 min) followed by 60 min incubation of iron compounds. Data were normalized to total protein in each well and are presented as percentage of untreated control (No Fe) ± Sdev, 6 cell replicates. An asterisk indicates a significant difference from the iron control, p < 0.5. **(B)** Total cellular iron load in chlorpromazine (10 μM; 30 min) and iron (0.5 mM, 60 min) treated cells measured by GFAAS. Iron content was normalised to total cellular protein (mg Fe/g protein). Data are means ± Sdev, n = 2, 4 cell replicates. The same letter (a, a and b, b) indicates a significant difference between the untreated control cells (No Fe) and iron treatment (a) and between treatments (b) (p < 0.05).

Preliminary data from previous studies suggested that iron from ferrous fumarate could be absorbed by clathrin-mediated endocytosis in Hutu-80 cells, which was confirmed in both post-confluent (14 days in culture) and non-confluent cells (48 h in culture) in the present study. In post-confluent cells, iron load (ferritin L production) was decreased after pre-treating the cells with a clathrin-mediated endocytosis inhibitor (Chlorpromazine, 100 μM) in the presence of ferrous fumarate (0.5 mM, 60 min), Figure 2A (61% ± 10%, 6 cell replicates, p = 0.000025). In addition, the effect on the ferritin H subunit was evaluated by Western blot analyses, Figure 2B, which was observed to decrease (39.5% ± 17%, n = 4, p = 0.009). In pre-treated non-confluent cells (Chlorpromazine, 10 μM), the iron load (ferritin L) was also decreased (37% ± 9%, 6 cell replicates, p = 0.0025), Figure 3A. In a pilot study, total iron content in the non-confluent cells was measured by GFAAS, Figure 3B. Iron uptake from a 1-h Ferrous fumarate dose contributed with a significant increase in total cellular iron load (23%, n = 2, 4 cell replicates p = 0.04). In endocytosis-inhibited ferrous fumarate treated cells, the total iron load was significantly decreased (24%, n = 2, 4 cell replicates, P = 0.009) compared to the ferrous fumarate control. There was no difference in total cellular iron load between endocytosis-inhibited ferric EDTA treated cells and the ferric EDTA controls (Figures 3A,B).

### 3.3 Membrane-asscoiated iron load in DMT1-Silenced vs. endocytosis-inhibited Hutu-80 cells estimated by ToF-SIMS

**FIGURE 4:**
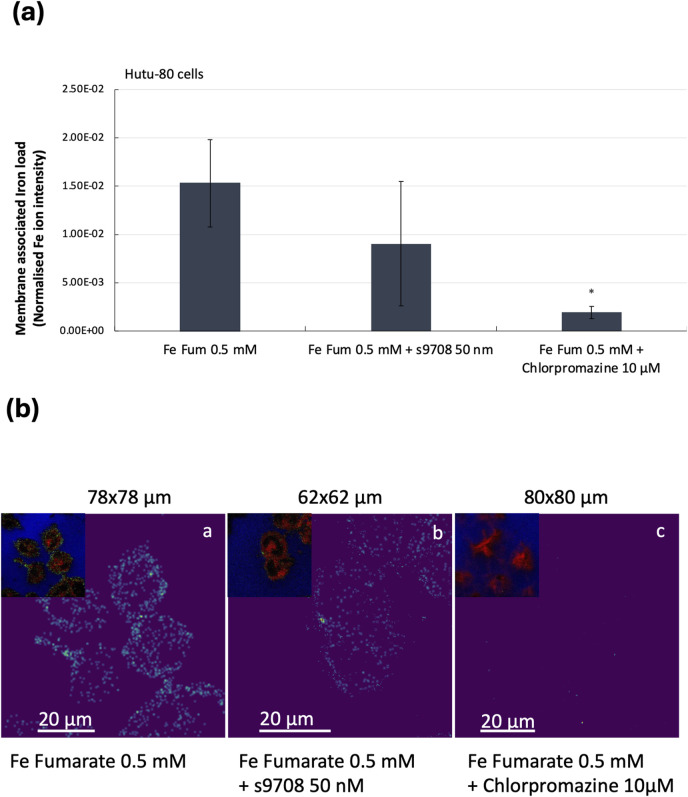
**(A)** Relative quantification of membrane-associated (100 nm depth) iron content in Hutu-80 cells as measured by the Iron ^56^Fe peak area in a ToF-SIMS image data set. Error bars indicate Standard error of the mean (SEM) between the iron controls and iron + inhibitors. Sample volume: Fe fumarate (iron controls); n = 2, 4 cell replicates, Fe fumarate and s9708; n = 2, 3 cell replicates, Fe fumarate and Chlorpromazine; n = 2, 4 cell replicates). **(B)** ToF-SIMS ion image overlays in RGB, showing membrane-associated iron in Hutu-80 cells (depth: 100 nm) treated with **a)** Fe fumarate ([Fe] = 0.5 mM). **b)** Fe fumarate ([Fe] = 0.5 mM) and siRNA s9708 (50 nM), **c)** Fe fumarate ([Fe] = 0.5 mM) and Chlorpromazine 10 μM. The upper left corner in each picture shows the phosphatidylcholine headgroup fragment at *m/z* 86 in red, Iron at *m/z* 56 in green and the Si signal from the glass at *m/z* 28 in blue. Field of view for (a) is 78 × 78 μm^2^ (b) 62 × 62 μm^2^ and (c) 80 × 80 μm^2^.

To confirm the findings that iron from ferrous fumarate can be absorbed by both DMT1 and by clathrin-mediated endocytosis, we measured the membrane-associated iron load using mass spectrometry imaging. Time of flight secondary ion mass spectrometry (ToF-SIMS) 3D profiling was used to probe the membrane-associated iron content by removing the outmost cell membrane (100 nm). The iron content was measured as the peak area of ^56^Fe normalised to the total value of the C_5_H_12_N ion fragment, *m/z* 86, from the phosphatidylcholine and phosphatidylserine headgroups. The reduced membrane-associated iron load in the DMT1-silenced cells (across n = 3 cell replicates, n = 2) could not be confirmed, Figure 4A (due to large standard deviations, it was not significantly different from the control cells). Endocytosis-inhibited Hutu-80 cells incubated with Fe fumarate ([Fe] = 0.5 mM) showed a significant reduction in membrane-associated iron load, 87.4%, p = 0.046, n = 2, 4 cell replicates, compared to ferrous fumarate controls, Figure 4A. Thus, confirming the trend in Figures 2, 3. However, direct comparisons of the percentage extent of inhibition should be avoided due to the variation in experimental conditions and endpoints. ToF-SIMS ion images from the Hutu-80 cells incubated with Fe fumarate confirmed the iron localization to mainly the outer parts of the cell membranes as can be seen in Figure 4B.

## 4 Discussion

Recent research indicates that various forms of supplemental iron, including chelates, salts, and nanoparticles, exert distinct effects on intracellular pathways associated with growth and the expression of inflammatory mediators. For example, our findings demonstrate that iron chelates, specifically ferric pyrophosphate and ferric EDTA, increase the expression of the oncogenic growth factor Amphiregulin in intestinal cell lines (Scheers et al., 2018). In contrast, simple iron salts such as ferrous fumarate and ferrous sulfate do not exhibit this effect. Across all studies conducted, ferrous fumarate consistently emerges as the form of iron that induces the least adverse cellular effects. Furthermore, it has been observed that ferric pyrophosphate and ferric EDTA, both high-affinity chelators, stimulate the production of prostaglandin E2 (PGE2) (Tarczykowska et al., 2023). Similarly, ferric nitrilotriacetate has been linked to elevated PGE2 production in rabbits (Okazaki et al., 1981). These findings suggest that the differential effects of various iron forms may depend on their mechanisms of absorption (uptake) and subsequent intracellular localization.

We hypothesize that iron from chelates is transported across the plasma membrane by Divalent Metal Transporter 1 (DMT1), due to the strong coordinate covalent bonds that maintain iron solubility. This solubility facilitates reduction and subsequent transport in ionic form. Notably, our study did not observe significant iron uptake from ferric EDTA via endocytosis. However, DMT1 knockdown experiments suggest that ferrous sulfate and ferrous fumarate are also transported by DMT1, indicating the involvement of additional mechanisms. An intriguing question arises as to whether the uptake mechanism determines whether released iron enters the cytosol as part of the labile iron pool or is chaperoned to intracellular or extracellular compartments.

During the development of the DMT1 silencing protocol, Western blot analysis revealed differences in the molecular weight of DMT1 between Hutu-80 cells and Caco-2 cells (Supplementary Figure S1). Specifically, DMT1 in Caco-2 cells exhibited an average molecular weight of 85 kDa, whereas in Hutu-80 cells it averaged 65 kDa. Previous research by Hubert and Hentze (2002) showed that Caco-2 cells predominantly express the DMT1 1A isoform. However, no literature data on DMT1 isoforms in Hutu-80 cells was found. A study by Mackenzie et al. estimated molecular weights for DMT1 isoforms as follows: 1A/IRE+/-isoforms at approximately 73 kDa and 1B/IRE+/-isoforms at approximately 61 kDa (Mackenzie et al., 2007). All these isoforms were functional; however, it was observed that 1A/IRE+/-isoforms are translated more efficiently than their 1B counterparts. Based on our Western blot data, we hypothesize that Hutu-80 cells predominantly express the DMT1 1B isoform. This hypothesis is supported by the lower DMT1 protein levels observed in Hutu-80 cells compared to Caco-2 cells (post-confluent with duodenal characteristics). Further investigation is required to confirm this hypothesis, such as removing attached sugars or other post-translational modifications to determine the actual molecular weight of DMT1.

It is noteworthy that DMT1 1A isoforms are highly expressed at the apical surface of polarized cells, whereas DMT1 1B isoforms have been reported to localize to early endosomes in various cell types (Tabuchi et al., 2002). This subcellular localization suggests that DMT1 1B may facilitate vesicular iron transport into the cytosol. Given these differences in subcellular localization and our hypothesis regarding Hutu-80 cells’ predominant expression of DMT1 1B isoforms, it is plausible that iron uptake via endocytosis is favored in Hutu-80 cells. This is further supported by Time-of-Flight Secondary Ion Mass Spectrometry (TOF-SIMS) data showing an 87.4% reduction in membrane-associated iron load when a clathrin lattice assembly inhibitor was present.

In conclusion, it is significant that ferrous fumarate does not induce an increase in oncogenic Amphiregulin or other pro-inflammatory mediators while being absorbed via endocytosis to a considerable extent. Future studies should focus on elucidating cytosolic iron transport mechanisms while confirming the specific import pathways involved during uptake. The current findings suggest that multiple mechanisms operate simultaneously; therefore, future research should aim to understand their relative contributions rather than focusing on a single uptake mechanism.

## Data Availability

The raw data supporting the conclusions of this article will be made available by the authors, without undue reservation.

## Supplementary material

SUPPLEMENTARY FIGURE S1
**(A)** Western blot of DMT1 in confluent non-treated Hutu-80 and Caco-2 cells. The average size of DMT1 (possibly including post-translational modifications) in Hutu-80 cells was 65 kD while it was 85 kD in Caco-2 cells. Upper and lower images show the same blot but with different exposure times (29 s for DMT1 and 5 s for GAPDH). **(B)** Western blot of DMT1 protein expression in confluent Hutu-80 and Caco-2 cells, treated with 200 and 1,000 nM of Silencer® Select siRNA s9708 (Invitrogen) for 48 h. 7.5 μg of total protein was loaded to each well.
