# Supplementary figures and images for: Uptake of iron from ferrous fumarate can be mediated by clathrin-dependent endocytosis in Hutu-80 cells
Source: Front Mol Biosci. 2025 Jan 27;12:1460565. doi: 10.3389/fmolb.2025.1460565 (PMC11807817; doi:10.3389/fmolb.2025.1460565)

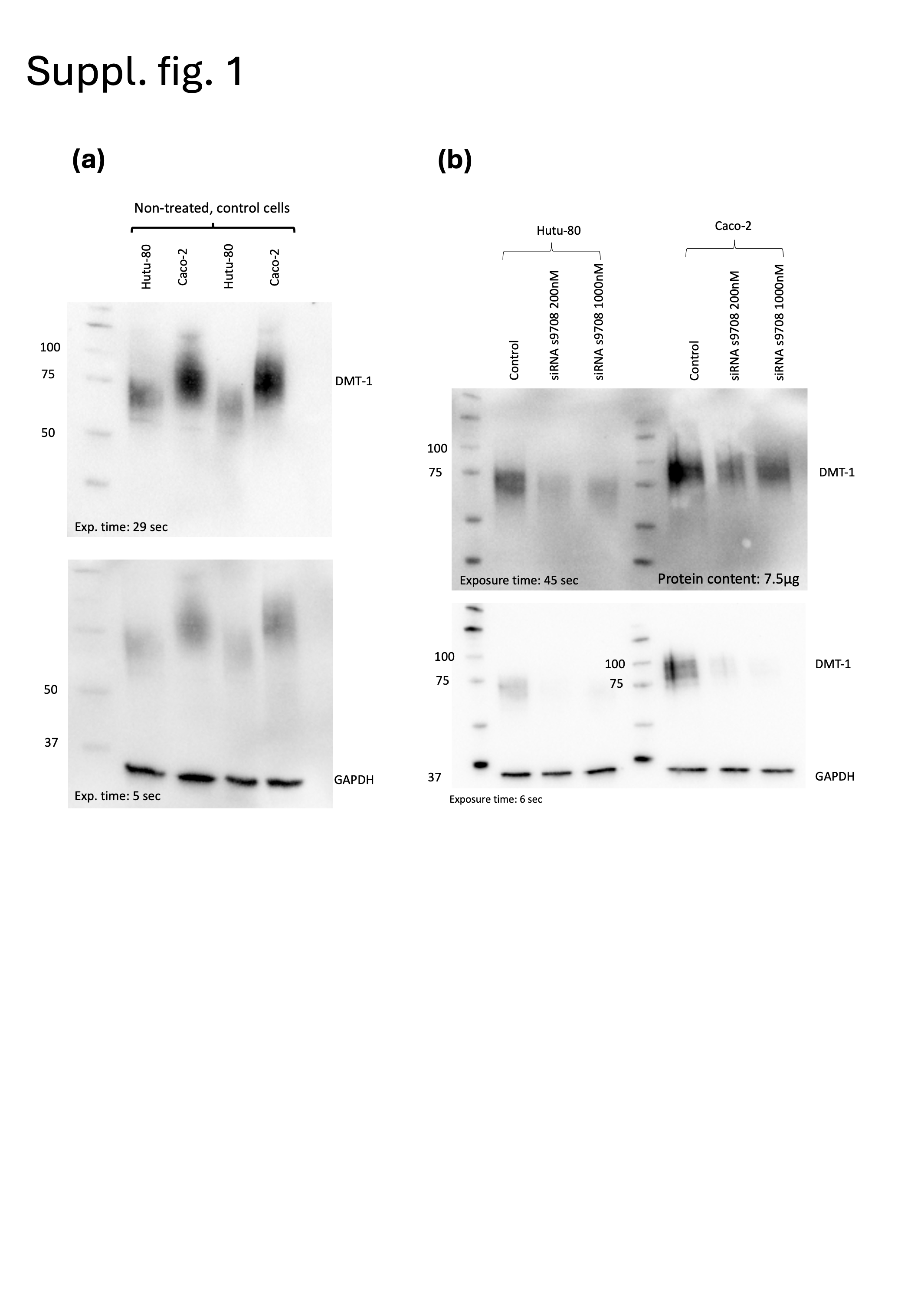

Supplement: Supplementary file 1 [file Image1.tiff]
