# Supplementary material for: Uptake of iron from ferrous fumarate can be mediated by clathrin-dependent endocytosis in Hutu-80 cells
Source: Front Mol Biosci. 2025 Jan 27;12:1460565. doi: 10.3389/fmolb.2025.1460565 (PMC11807817; doi:10.3389/fmolb.2025.1460565)
Supplement: Supplementary file 2 [file Table1.docx]

Table S1. GFAAS temperature program and analysis conditions.

| Step | Temp [^o^C] | Time [s] | Gas flow [L/min] |
| --- | --- | --- | --- |
| 1 | 85 | 5.0 | 0.3 |
| 2 | 95 | 30.0 | 0.3 |
| 3 | 120 | 10.0 | 0.3 |
| 4  5  6 | 700  700  700 | 5.0  1.0  2.0 | 0.3  0.3  0.0 |
| 7  8  9 | 2300  2300  2300 | 0.8  2.0  2.0 | 0.0  0.0  0.3 |
|  | | | |
